# Quantitative assessment of myocardial blood flow in coronary artery disease by cardiovascular magnetic resonance: comparison of Fermi and distributed parameter modeling against invasive methods

**DOI:** 10.1186/s12968-016-0270-1

**Published:** 2016-09-13

**Authors:** Giorgos Papanastasiou, Michelle C. Williams, Marc R. Dweck, Shirjel Alam, Annette Cooper, Saeed Mirsadraee, David E. Newby, Scott I. Semple

**Affiliations:** 1 Clinical Research Imaging Centre, University of Edinburgh, Edinburgh, UK; 2 Centre for Cardiovascular Science, University of Edinburgh, Edinburgh, UK

**Keywords:** Cardiovascular magnetic resonance, Myocardial blood flow, Fermi modeling, Distributed parameter modeling, Invasive methods

## Abstract

**Background:**

Mathematical modeling of perfusion cardiovascular magnetic resonance (CMR) data allows absolute quantification of myocardial blood flow and can potentially improve the diagnosis and prognostication of obstructive coronary artery disease (CAD), against the current clinical standard of visual assessments. This study compares the diagnostic performance of distributed parameter modeling (DP) against the standard Fermi model, for the detection of obstructive CAD, in per vessel against per patient analysis.

**Methods:**

A pilot cohort of 28 subjects (24 included in the final analysis) with known or suspected CAD underwent adenosine stress-rest perfusion CMR at 3T. Data were analysed using Fermi and DP modeling against invasive coronary angiography and fractional flow reserve, acquired in all subjects. Obstructive CAD was defined as luminal stenosis of ≥70 % alone, or luminal stenosis ≥50 % and fractional flow reserve ≤0.80.

**Results:**

On ROC analysis, DP modeling outperformed the standard Fermi model, in per vessel and per patient analysis. In per patient analysis, DP modeling-derived myocardial blood flow at stress demonstrated the highest sensitivity and specificity (0.96, 0.92) in detecting obstructive CAD, against Fermi modeling (0.78, 0.88) and visual assessments (0.79, 0.88), respectively.

**Conclusions:**

DP modeling demonstrated consistently increased diagnostic performance against Fermi modeling and showed that it may have merit for stratifying patients with at least one vessel with obstructive CAD.

**Trial Registration:**

Clinical Trial Registration: Clinicaltrials.gov NCT01368237

Registered 6 of June 2011. URL: https://clinicaltrials.gov/ct2/show/NCT01368237

**Electronic supplementary material:**

The online version of this article (doi:10.1186/s12968-016-0270-1) contains supplementary material, which is available to authorized users.

## Background

Perfusion cardiovascular magnetic resonance (CMR) is a technique that allows the non-invasive assessment of coronary artery disease (CAD) [1, 2]. Clinically, the current standard method of assessment of perfusion CMR is based either on visual estimates of the images, or on a semi-quantitative assessment of perfusion index [3, 4]. Visual assessments of areas of abnormal perfusion rely on the presence of myocardial areas with normal perfusion for direct comparison. Visual estimates are particularly difficult in multi-vessel disease where there may be minimal areas of normal perfusion to compare against, or in cases of severe left ventricular impairment [5] in which slow bolus dispersion leads to low contrast enhancement globally within the myocardium. Mathematical modeling of perfusion imaging data allows absolute quantification of myocardial blood flow (MBF) and it may be particularly useful in cases in which visual assessment is compromised. By quantifying perfusion, it also has the potential to minimize interobserver variability and to improve the diagnosis and prognostication of CAD [5, 6].

Fermi deconvolution modeling is an empirical-mathematical model used to estimate MBF from perfusion CMR data during first-pass of gadolinium-based extracellular contrast agents [7]. Distributed parameter (DP) deconvolution modeling is based upon physiological principles of tracer kinetics analysis and it can provide MBF quantification and additional information about coronary vascularity and permeability [8]. This includes estimates of intravascular space, extravascular-extracellular space, permeability surface area product, extraction fraction and volume of distribution.

Fermi modeling is currently the most popular approach for quantitative analysis of perfusion CMR data [5]. The diagnostic performance of Fermi modeling in detecting obstructive CAD has been assessed, with variable conclusions whether it provides additive clinical information to visual assessment [9] or not [5, 10]. In contrast to Fermi modeling, the diagnostic performance of DP modeling has not at this point been assessed against invasive clinical standard methods. In addition, there is still a disagreement whether Fermi modeling can provide haemodynamic thresholds either in per vessel [5, 10] or in per patient [11] basis, for robust patient stratification in the presence of obstructive CAD. A model comparison in per vessel against per patient analysis has not been fully assessed yet in a single CMR study.

The objective of this study was two-fold. Firstly, to investigate whether either Fermi or DP modeling may be more accurate in detecting reduced MBF in obstructive CAD, when compared against the current invasive clinical standard assessment of invasive coronary angiography and fractional flow reserve in a pilot population. The second objective of this study was to assess the diagnostic performance of both models in per vessel against per patient based analysis in this pilot population.

## Methods

### Study population and design

28 patients with history of stable angina and with known or suspected CAD were recruited for perfusion CMR. Exclusion criteria for all subjects included history of severely compromised renal function (Glomerular filtration rate ≤ 30  mL/min), pregnancy and contraindications to CMR. The study was performed with the approval of the institutional research ethics committee, in accordance with the Declaration of Helsinki and with the written informed consent of all subjects. All subjects were instructed to abstain from caffeine for 12 h before CMR. All patients underwent invasive coronary angiography and fractional flow reserve.

### Cardiac magnetic resonance imaging

Perfusion CMR was acquired using a 3T Verio imaging system (Siemens, Healthcare Gmbh, Erlangen, Germany) using electrocardiogram-gating, as previously described [12]. Standard cardiac imaging planes and a short axis stack of left ventricular cine data were acquired using routine steady state free precession (TrueFISP) acquisitions. T1 MOLLI maps were acquired using the Siemens Works in Progress Package #448, Quantitative Cardiac Parameter Mapping [13]. Stress imaging was performed by intravenously administering 140 μg/kg/min of adenosine (Adenoscan, Sanofi Aventis) for 4 min and confirmation of patient symptoms. Fifty dynamic perfusion images were obtained at diastole across three short-axis view slices, covering 16 of the standard myocardial segments [14]. A turbo-fast low angle shot saturation recovery prepared single-shot gradient echo pulse sequence was used with imaging parameters: repetition time/ echo time 2.20 ms/1.07 ms, flip angle 12^o^, slice thickness 8 mm, preparation pulse delay to central line of k-space 100 ms, matrix size 192 × 108 and FoV 330 mm x 440 mm. With the application of GRAPPA (accelerator factor of 3) and partial Fourier acquisition of 0.75, each dynamic frame consisted of 48-phase encoding lines.

An intravenous bolus of 0.05 mmol/kg of a gadolinium-based contrast agent (Gadovist, Bayer Healthcare) was injected at 4 mL/s using an MR-compatible pump injector (Spectris Solaris, Medrad, Bayer). To allow clearance of residual contrast agent, rest perfusion imaging was performed 15 min after the adenosine-stress scan by repeating the same acquisition protocol in all subjects.

### Invasive coronary angiography and fractional flow reserve

All patients underwent invasive coronary angiography and fractional flow reserve at the Royal Infirmary of Edinburgh. Fractional flow reserve was assessed for major epicardial vessels and defined as the ratio between distal coronary pressure and aortic pressure measured simultaneously at maximal adenosine-induced (intravenous 140 μg/kg/min) hyperaemia [15, 16]. Haemodynamically significant (obstructive) CAD was defined as luminal stenosis ≥70 % on invasive coronary angiography, or fractional flow reserve <0.80 and luminal stenosis ≥50 %. Outcomes from the three main coronary vessels were classified into 2 groups: Group 1, (no, minor or non)-obstructive CAD with luminal stenosis <50 % or with luminal stenosis ≥50 % and fractional flow reserve > 0.80; Group 2, obstructive CAD with luminal stenosis of ≥70 % alone, or luminal stenosis ≥50 % and fractional flow reserve ≤0.80 [15, 16].

### Visual analysis

Perfusion CMR images were analyzed by 2 experienced observers blinded to all other data. The perfusion CMR scans were reported for the three main epicardial vessel territories and classified as positive for obstructive CAD in the presence of a stress-induced perfusion defect which was transmural and/or involved ≥ 1 myocardial segment, corresponding to the maximum sensitivity and specificity [10]. In the event of disagreement, the images were reviewed together and a consensus was reached.

### Quantitative CMR analysis

Endocardial and epicardial MR contours were outlined using dedicated cardiac image analysis software (QMass, Medis, The Netherlands) to generate a standardised 16-segment American Heart Association (AHA) model of the heart (reference marker for myocardial segmentation was placed in the anteroseptal conjunction of the left and right ventricle) [14]. Quantification of MBF was performed using customised in-house software developed in Matlab (MathWorks Inc., Natick, MA), as previously described [12]. Myocardial and arterial input function signal intensity-time curves were converted to gadolinium concentration-time curves using the method of Larsson et al (see details in Additional file 1) [17]. Model-dependent deconvolution analysis was implemented to measure MBF using Fermi and 1-barrier 2-region DP functions as previously described (see details and functions in Additional file 2) [12]. To account for the delay time between the onset of contrast enhancement in the arterial input function and the myocardium, both models were fitted to the data multiple times, from zero to six times the temporal resolution at dynamic perfusion acquisition. The delay time reaching the optimal x^2^ fit to the data was used in the analysis [11, 12]. In DP modeling, additional microvascular characteristics were also calculated (Additional file 3). Myocardial perfusion reserve (MPR) was calculated by dividing the hyperemic MBF by the resting flow. The mean myocardial perfusion reserve of the two lowest scoring myocardial segments (MPR_2_) was also calculated for each vessel territory and its accuracy in detecting obstructive CAD was examined [5, 10]. Both models were applied to each of the 16 AHA segments. MBF, MPR and MPR_2_ were then averaged per epicardial vessel territory (vessel territories corresponded to the three main coronary vessels, also defined according to the 16 segment AHA model [14]). Mean values for MBF at stress, MPR and MPR_2_ were classified accordingly for per vessel and per patient based analysis (see classification in the results section referring to visual MR analysis).

### Statistical analysis

Dedicated software were used for statistical analysis (R Foundation for statistical computing, Vienna, Austria, Analyse-it, Analyse-it Software, Leeds, England). Receiver-operating characteristic (ROC) analysis was used to determine threshold values for absolute MBF at stress, MPR and MPR_2_ with the greatest sensitivity and specificity to detect obstructive CAD (Group 2 versus Group 1). The maximal Youden Index was used to determine the optimal threshold values [5, 10]. The area under the curve (AUC) was calculated using trapezoidal numerical integration and a Delong et al nonparametric comparison was used to compare the diagnostic performance of quantitative methods [18]. Bland Altman plots were used to investigate systematic bias between Fermi and DP modeling values for MBF, MPR and MPR_2_.

An interobserver reliability analysis was performed for visual estimates using Cohen kappa statistic. Statistical differences in MBF values and in myocardial perfusion ratios between patient Groups (Group 2 against Group 1), were investigated by implementing a two sample *t*-test. Statistical significance was defined as two-sided *P* value < 0.05.

## Results

### Patients

The baseline demographics of the patient cohort are presented in Table 1. In the final analysis, data from 24 patients were used. Quantitative perfusion analysis was performed in 72 vessel territories in total. 3 patients did not complete the imaging session due to claustrophobia. 1 patient was excluded from the analysis due to electrocardiographic failures which caused considerable through plane motion.

**Table 1:** Baseline demographics

Parameter	Data (*n* = 24)
Age (yrs)	63 ± 7
Male	20 (83)
BMI	29 ± 5
Hypertension	13 (54)
Hct	0.43 ± 0.02
Diabetes	
Type 1	0 (0)
Type 2	3 (13)
Angina	23 (96)
NSTEMI	4 (17)
STEMI	3 (13)
PVD	0 (0)
CVD	2 (8)
Smoking	
Current	6 (25)
Previous	15 (63)
PCI	4 (17)
Medication	
Statin	21 (88)
Beta-blocker	20 (83)
Angiographic data (per vessel)	
Group 1	46 (64)
Group 2	26 (36)

Parentheses show (%). *BMI* Body mass index, *Hct* haematocrit, *NSTEMI* non-ST segment elevation myocardial infarction, *STEMI* ST segment elevation myocardial infarction, *PVD* peripheral vascular disease, *CVD* cardiovascular disease, *PCI* percutaneous coronary intervention

All patients underwent invasive coronary angiography and fractional flow reserve assessment. 16 patients (67 %) had at least 1 vessel territory classified with obstructive CAD (Group 2). 10 patients had 1-vessel disease, 2 had 2-vessel disease and 4 had 3-vessel disease.

### Visual CMR analysis

**Fig. 1:**
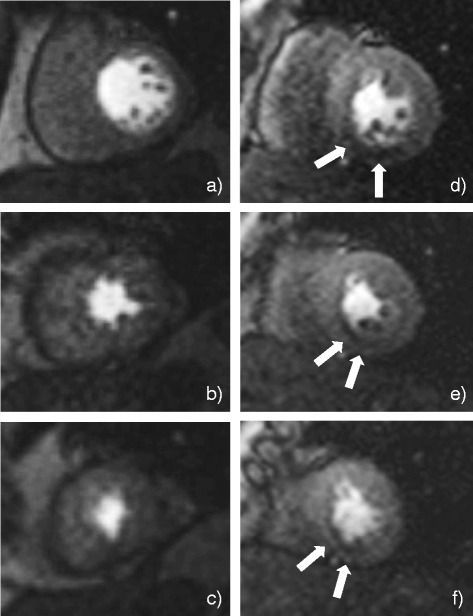
Perfusion CMR images from a patient with minor CAD (**a**, **b**, **c**) and a patient with (1-vessel) right coronary artery disease (**d**, **e**, **f**). White arrows show perfusion defect in the inferior and inferoseptal myocardial regions. Basal (**a**, **d**), mid-ventricular (**b**, **e**) and apical slices (**c**, **f**) are shown

The interobserver variability was kappa = 0.81 (95 % CI: 0.73 to 0.89). In per vessel analysis, vessel classification occurred using the cut off criteria described in the methods section for invasive assessments. In per patient analysis, patients with all vessel territories identified with (no, minor or non)- obstructive CAD were classified in Group 1, whilst patients with at least one vessel detected with obstructive CAD, were classified in Group 2. Based on these criteria (applied for both visual and quantitative CMR analysis), diagnostic performance (i.e. sensitivity, specificity, positive predictive value and negative predictive value) in per vessel and per patient visual MR analysis against invasive methods, are presented in Table 2. Examples of MR perfusion images are presented in Fig. 1.

**Table 2:** Diagnostic performance of visual CMR estimates

Visual estimates from MR	Per vessel	Per patient
Sensitivity	0.73 (0.50, 0.88)	0.79 (0.49, 0.94)
Specificity	0.80 (0.64, 0.89)	0.88 (0.47, 0.99)
PPV	0.64 (0.43, 0.81)	0.92 (0.60, 0.99)
NPV	0.85 (0.70, 0.94)	0.70 (0.35, 0.92)

Sensitivity, specificity, PPV, NPV is shown in per vessel and per patient analysis. Parentheses show (95 % confidence intervals). *MR* magnetic resonance, *PPV* positive predictive value, *NPV* negative predictive value

### Quantitative CMR analysis

**Fig. 2:**
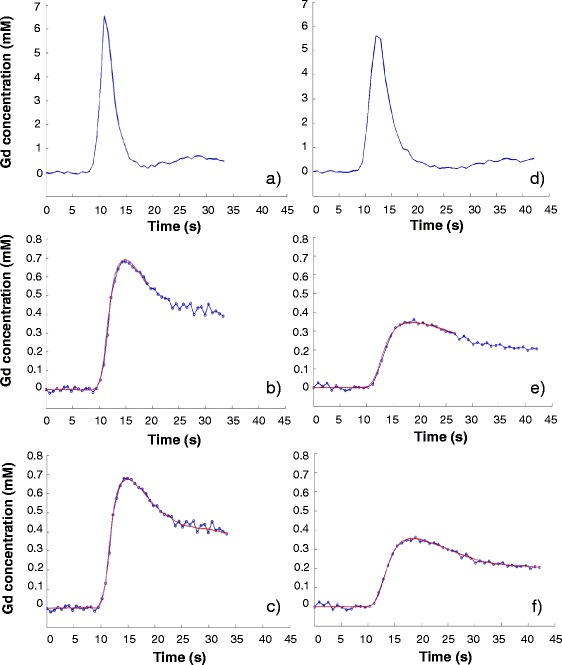
Arterial input functions and model fits on myocardial tissue curves from a patient with minor (**a**, **b**, **c**) and a patient with 1-vessel (**d**, **e**, **f**) CAD. **a**, **d**, **b**, **e**, **c**, f show arterial input functions, Fermi modeling and distributed parameter modeling fits, respectively. Gd: gadolinium

Initially, quantitative MBF analysis was performed on a per vessel basis. Examples of arterial input function and model fitting for both models are shown in Fig. 2. Mean values for all Fermi- and DP modeling-derived haemodynamic parameters (i.e. absolute MBF at stress, MPR and MPR_2_), for both Groups 1 and 2, are presented in Table 3.

**Table 3:** Mean (SD) values for MBF at stress, MPR and MPR_2_, for Groups 1 and 2

Haemodynamic parameter-Model	Per vessel		Per patient	
	Group 1	Group 2	Group 1	Group 2
MBF Fermi (mL/min/mL)	2.49 (0.96)	1.96 (0.66)	3.07 (0.71)	1.97 (0.75)
MBF DP (mL/min/mL)	2.01 (0.71)	1.36 (0.39)	2.53 (0.37)	1.41 (0.46)
MPR Fermi	2.09 (0.85)	1.55 (0.59)	2.61 (0.76)	1.61 (0.59)
MPR DP	1.78 (0.71)	1.12 (0.38)	2.26 (0.61)	1.21 (0.39)
MPR2 Fermi	1.79 (0.81)	1.08 (0.45)	2.24 (0.77)	1.19 (0.49)
MPR2 DP	1.53 (0.65)	0.86 (0.31)	1.97 (0.56)	0.95 (0.32)

Mean values in per vessel and per patient based analysis. *MBF* myocardial blood flow, *MPR* myocardial perfusion reserve, *MPR*_*2*_ myocardial perfusion reserve of the two lowest scoring segments, *DP* distributed parameter modeling

Table 4 demonstrates results for each model. Significant differences in haemodynamic parameter values between Groups 1 and 2 were considerably higher for DP modeling, compared to Fermi modeling (Table 4). No differences were observed for blood flow values at rest between Groups 1 and 2, for both models. Significant differences were observed in all DP modeling-derived microvascular characteristics, between Groups 1 and 2 (all *P* ≤0.01). Mean values for microvascular characteristics are also presented (Additional file 4).

**Table 4:** Results from t-test and ROC analysis in per vessel and per patient based analysis

Statistical analysis/ Model	Per vessel		Per patient	
	Fermi	DP	Fermi	DP
*P* values from t-test comparisons				
MBF (G1 vs G2)	0.02*	<0.0001 †	<0.00001*	<0.00001 †
MPR (G1 vs G2)	<0.01*	<0.0001 †	<0.00001*	<0.00001 †
MPR2 (G1 vs G2)	<0.00001*	<0.00001 †	<0.00001*	<0.00001 †
AUCs from ROC analysis				
MBF	0.68 (0.55, 0.80)	0.76 (0.65, 0.87)	0.86 (0.77, 0.94)	0.97 (0.92, 1.00)
MPR	0.69 (0.56, 0.81)	0.79 (0.68, 0.89)	0.85 (0.75, 0.94)	0.94 (0.87, 1.00)
MPR_2_	0.77 (0.66, 0.88)	0.82 (0.72, 0.92)	0.88 (0.80, 0.96)	0.96 (0.91, 1.00)
Thresholds on ROC analysis				
MBF (mL/min/mL)	2.49	1.75	2.60	2.00
MPR	1.76	1.45	1.88	1.59
MPR_2_	1.36	1.07	1.49	1.47
*P* values from comparisons of ROC curves between haemodynamic parameters				
MBF vs MPR	0.81	0.54	0.80	0.35
MBF vs MPR_2_	0.04*	0.19	0.45	0.68
MPR vs MPR_2_	0.0022*	0.20	0.17	0.33
Difference in AUC of ROC curves between haemodynamic parameters				
MBF vs MPR	-0.01 (-0.10, 0.08)	-0.03 (-0.11, 0.05)	0.01 (-0.06, 0.08)	0.03 (-0.03, 0.09)
MBF vs MPR_2_	-0.09 (-0.18, 0.01)	-0.06 (-0.14, 0.02)	-0.03 (-0.10, 0.04)	0.01 (-0.04, 0.06)
MPR vs MPR_2_	-0.08 (-0.16, 0.01)	-0.03 (-0.09, 0.02)	-0.04 (-0.09, 0.01)	-0.02 (-0.06, 0.02)

Statistically significant differences are indicated with * and † († show at least two orders of magnitude smaller P values compared to *, both in per vessel and per patient analysis). Parentheses show (95 % confidence intervals). *MBF* myocardial blood flow, *MPR* myocardial perfusion reserve, *MPR*_*2*_ myocardial perfusion reserve of the two lowest scoring segments, *AUC* area under the curve, *DP* distributed parameter modeling, *G1* Group 1, *G2* Group 2

Systematic bias between Fermi- and DP modeling-derived values was investigated using Bland Altman plots (Additional file 5). The average bias was calculated as the Fermi modeling-derived estimates minus the DP modeling-derived estimates across all three haemodynamic parameters. For MBF at stress, MPR and MPR_2_, the average bias (95 % CI) was 0.55 (-0.51, 1.62), 0.38 (-0.54, 1.31), 0.25 (0.51, 1.00), respectively.

**Fig. 3:**
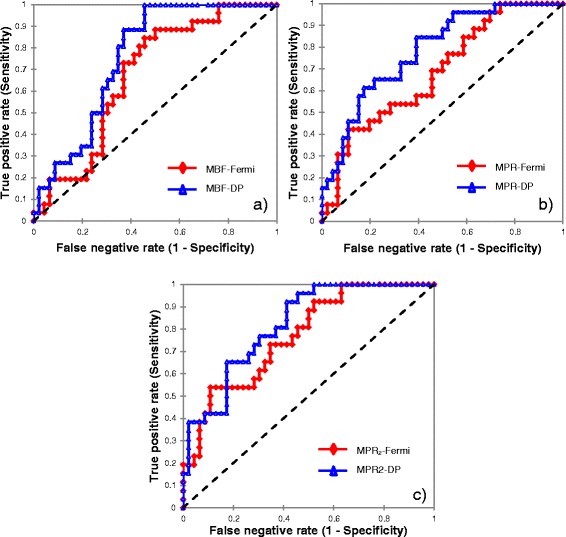
ROC curves presenting diagnostic performance for both models in per vessel analysis with (**a**), (**b**) and (**c**) showing measures of myocardial blood flow at stress, myocardial perfusion reserve and myocardial perfusion reserve of the two lowest scoring segments, respectively

**Fig. 4:**
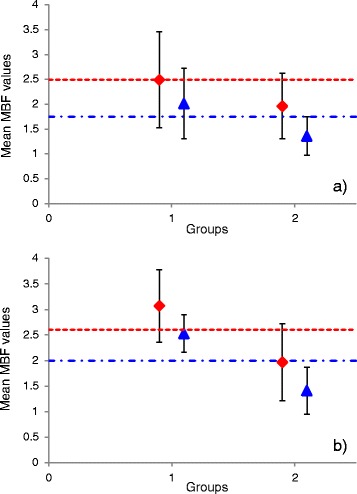
Mean myocardial blood flow values (SD) at stress in per vessel (**a**) and per patient (**b**) analysis. Mean values and thresholds for Fermi and distributed parameter modeling are represented with red diamond, red square dot line and blue triangle, blue dash dot line, respectively. MBF: myocardial blood flow

ROC analysis graphs are illustrated in Fig. 3. Haemodynamic thresholds defined on ROC analysis (Table 4) as compared to mean values for MBF at stress are illustrated in Fig. 4a. The AUC for MPR_2_ was significantly higher compared to both MBF at stress and MPR for Fermi modeling (Table 4). There were no significant differences between ROC curves across all three haemodynamic parameters for DP modeling (Table 4).

Table 5 shows comparisons on ROC analysis between Fermi and DP modeling across all three haemodynamic parameters. Significant differences were observed in AUC between Fermi and DP modeling, except for the case of MPR_2_ (Table 5). The diagnostic performance was consistently higher for DP modeling, compared to Fermi modeling (Table 6).

**Table 5:** Results from ROC analysis for all Fermi versus DP modeling comparisons

Statistics	Fermi vs DP modeling (Per vessel)	Fermi vs DP modeling (Per patient)
*P* values from comparisons of ROC curves between models		
MBF	0.01*	0.0065*
MPR	0.02*	0.0089*
MPR_2_	0.21	0.0064*
Difference in AUC of ROC curves between models		
MBF	-0.08 (-0.15,-0.02)	-0.11 (-0.19,-0.03)
MPR	-0.10 (-0.18,-0.02)	-0.09 (-0.16,-0.02)
MPR_2_	-0.05 (-0.13, 0.03)	-0.07 (-0.12,-0.02)

Statistically significant differences are indicated with *. Comparisons in per vessel and per patient analysis are presented. Parentheses show 95 % confidence intervals. *DP* distributed parameter modeling, *MBF* myocardial blood flow, *MPR* myocardial perfusion reserve, *MPR*_*2*_ myocardial perfusion reserve of the two lowest scoring segments, *AUC* area under the curve

**Table 6:** Diagnostic performance of Fermi and DP modeling in per vessel and per patient analysis

Haemodynamic parameter-Model	Sensitivity	Specificity	PPV	NPV
Fermi-MBF PV	0.85 (0.76 to 0.94)	0.54 (0.43 to 0.65)	0.51 (0.39 to 0.63)	0.86 (0.74 to 0.98)
DP-MBF PV	0.89 (0.79 to 0.99)	0.63 (0.52 to 0.74)	0.58 (0.48 to 0.68)	0.91 (0.84 to 0.98)
Fermi-MPR PV	0.69 (0.50 to 0.88)	0.54 (0.40 to 0.68)	0.46 (0.32 to 0.60)	0.76 (0.62 to 0.90)
DP-MPR PV	0.85 (0.72 to 0.98)	0.61 (0.48 to 0.74)	0.55 (0.43 to 0.67)	0.88 (0.76 to 1.00)
Fermi-MPR2 PV	0.73 (0.58 to 0.88)	0.65 (0.51 to 0.79)	0.54 (0.40 to 0.68)	0.81 (0.68 to 0.94)
DP-MPR2 PV	0.77 (0.62 to 0.92)	0.70 (0.56 to 0.84)	0.58 (0.46 to 0.70)	0.84 (0.75 to 0.94)
Fermi-MBF PP	0.78 (0.66 to 0.90)	0.88 (0.76 to 1.00)	0.93 (0.86 to 1.00)	0.66 (0.55 to 0.77)
DP-MBF PP	0.96 (0.91 to 1.00)	0.92 (0.85 to 0.99)	0.96 (0.91 to 1.00)	0.92 (0.84 to 1.00)
Fermi-MPR PP	0.69 (0.55 to 0.83)	0.83 (0.70 to 0.96)	0.90 (0.80 to 1.00)	0.58 (0.46 to 0.70)
DP-MPR PP	0.88 (0.76 to 1.00)	0.88 (0.78 to 0.98)	0.94 (0.88 to 1.00)	0.78 (0.66 to 0.90)
Fermi-MPR2 PP	0.80 (0.66 to 0.94)	0.79 (0.62 to 0.94)	0.89 (0.78 to 1.00)	0.66 (0.55 to 0.77)
DP-MPR2 PP	0.92 (0.86 to 0.98)	0.83 (0.74 to 0.92)	0.92 (0.86 to 0.98)	0.83 (0.73 to 0.93)

Parentheses show 95 % confidence intervals. *PPV* positive predictive value, *NPV* negative predictive value, *DP* distributed parameter modeling, *MBF* myocardial blood flow, *MPR* myocardial perfusion reserve, *MPR*_*2*_ myocardial perfusion reserve of the two lowest scoring segments, *PV* per vessel analysis, *PP* per patient analysis

Following per vessel analysis, quantitative MBF analysis was investigated on a per patient basis. Mean values for all three Fermi- and DP modeling-derived haemodynamic parameters for Groups 1 and 2 are presented in Table 3.

Significant differences in haemodynamic parameter values between Groups 1 and 2 increased further for both models, with DP modeling demonstrating consistently greater differences, compared to Fermi modeling (Table 4).

Haemodynamic thresholds on ROC analysis and mean values for MBF at stress in per patient analysis are demonstrated in Fig. 4b. The AUC were generally increased in per patient analysis, in comparison with per vessel analysis (Table 4). No significant differences were observed in ROC curves between haemodynamic parameters for either model, in per patient analysis (Table 4).

**Fig. 5:**
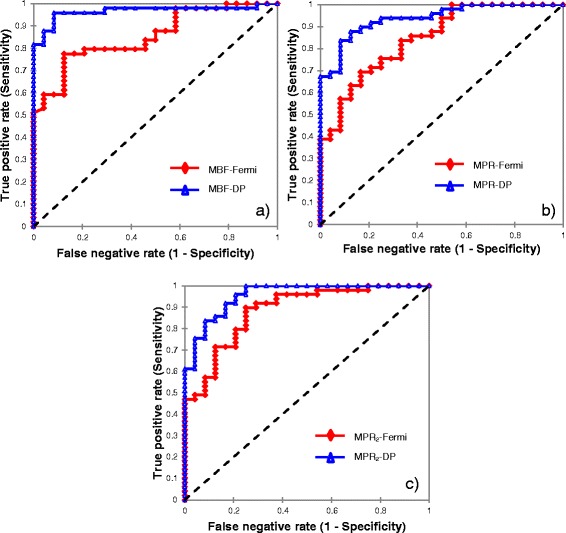
ROC curves demonstrating diagnostic performance for both models in per patient analysis with (**a**), (**b**) and (**c**) showing measures of myocardial blood flow at stress, myocardial perfusion reserve and myocardial perfusion reserve of the two lowest scoring segments, respectively

The AUC were significantly superior for DP modeling, compared to Fermi modeling ( Fig. 5, Table 5). The diagnostic performance of both models was increased in per patient analysis compared against per vessel analysis, with DP modeling outperforming Fermi modeling across all three haemodynamic parameters (Table 6).

## Discussion

The main findings of this study demonstrated that DP modeling outperformed the standard Fermi modeling in the setting of obstructive CAD detection, in both per vessel and per patient analysis. When compared with visual and quantitative CMR analysis, DP modeling-derived MBF at stress in per patient analysis, showed the highest diagnostic performance for the detection of obstructive CAD in our pilot population.

### Visual versus quantitative CMR analysis

The interobserver variability for visual analysis was similar to a previously published value [10] and higher compared to another study [9]. In per vessel based analysis, visual CMR estimates gave higher specificity compared to quantitative CMR estimates (Tables 2 versus 6). However, quantitative CMR analysis showed superior sensitivity using both Fermi and DP modeling-derived MBF at stress, compared to visual CMR assessments.

In per patient based analysis, DP modeling demonstrated superior diagnostic performance in detecting obstructive CAD, compared to visual CMR estimates (Tables 2 versus 6).

### Distributed parameter versus Fermi analysis

This is the first study assessing the diagnostic performance of 1-barrier 2-region DP modeling in 24 patients with known or suspected CAD, against invasive methods. Studies assessing the diagnostic performance of fully quantitative perfusion CMR methods in patients have mainly focused on the use of Fermi modeling [5, 9, 10, 19], or on a model-independent approach [20], for detecting obstructive CAD. A recent perfusion CMR study has assessed four different model applications (Fermi model, uptake model, one-compartmental model, model-independent approach) at 1.5T, in which it was shown that the diagnostic performance of quantitative MR analysis did not significantly differ between modeling methods, for obstructive CAD detection [11]. In our data analysis, the diagnostic power of Fermi modeling was in agreement with previously published studies [5, 9–11, 19]. However, DP modeling showed significantly higher diagnostic performance compared to Fermi modeling, which was consistent throughout both per vessel and per patient based analysis.

To date, no other perfusion CMR study has accurately identified perfusion abnormalities in the presence of significant CAD using only MBF value at stress as a measure (estimated per epicardial vessel territory). Perfusion CMR studies have instead defined the use of the lowest scoring segments for detecting obstructive CAD. This approach was applied either by using MPR as measure [5, 10, 19], or MBF at stress as measure [20], or both [11]. Patel et al. used Fermi modeling-derived MPR, to assess quantitative MR analysis in patients with obstructive CAD [9]. In our data, no differences were observed between all three haemodynamic parameters for either model, other than the case of the Fermi modeling derived-MPR_2_, which showed improvement against MBF at stress and MPR, in per vessel analysis. This agrees with previous studies demonstrating no difference between MBF at stress and MPR when the measure of the lowest scoring segments was examined [11, 20].

The optimal thresholds in per vessel (1.75 mL/min/mL) and in per patient (2.00 mL/min/mL) analysis for DP modeling-derived MBF at stress (Table 4), were in agreement with a previous positron emission tomography (PET) myocardial perfusion study (1.85 mL/min/mL) which aimed to localise perfusion defects to significantly stenotic coronary arteries in per vessel and per patient basis (≥70 % on invasive angiograms) [21]. It is important to note that PET is currently considered the reference standard for absolute non-invasive quantification of MBF [1, 5, 21].

The Fermi model demonstrated higher haemodynamic thresholds in both per vessel (2.49 mL/min/mL) and per patient (2.60 mL/min/mL) based analysis, compared to DP modeling (Table 4). Threshold values for Fermi modeling were in agreement with a previously published value (2.30 mL/min/mL) [22]. However, thresholds for Fermi modeling were in a slightly higher range compared to other PET perfusion studies (1.86, 2.50 and 2.45 mL/min/mL) [23–25] respectively, which aimed to identify perfusion abnormalities against lower angiographic thresholds (≥50 % on invasive angiograms). In our analysis, higher perfusion estimates were observed for Fermi modeling compared to DP modeling, across all three haemodynamic parameters (Table 3, Bland Altman plot analysis). In the same context, other perfusion studies demonstrated that the Fermi model estimates MBF values that were systematically increased compared to DP modeling [12, 26], to two-compartmental modeling, model-independent and Patlak model analysis [27], as well as against PET imaging data analysed with the Patlak model [5]. It is known that arterial input function saturation effects that may be present in single bolus data can result in significant overestimation of MBF in Fermi modeling [28]. Our group has previously demonstrated that the DP model can be less dependent on arterial input function saturation effects, compared to the Fermi model [12]. Any MBF overestimations using Fermi modeling may have become pronounced in some of our subjects due to saturation effects in our single bolus data. This may explain its lower sensitivity in detecting hypoperfusion in obstructive CAD (susceptible to false negatives), compared to DP modeling.

### Per vessel versus per patient quantitative analysis

The significant differences between (no, minor or non)-obstructive and obstructive CAD (Table 4 and 5), the AUC (Table 4, Figs. 3 and 5) and the diagnostic performance (Table 6) of both models were considerably superior in per patient analysis, compared to per vessel analysis. DP modeling-derived MBF at stress in per patient analysis demonstrated the highest diagnostic performance in detecting impaired haemodynamics in obstructive CAD (Table 6) and compares favourably against previous findings [9, 11]. These outcomes indicate that it may have merit for the stratification of patients with at least one vessel with obstructive CAD.

The specificities and positive predictive values in per vessel based analysis were in agreement with those reported by a previous study [19], but in a lower range compared to other investigations [5, 10]. Quantitative CMR analysis identified hypoperfusion in vessels with (no, minor or non)-obstructive CAD, which increased the false positives in per vessel analysis. 72 % of the patients with (no, minor or non)-obstructive disease had at least one vessel with obstructive CAD (Table 1). Also, the vast majority of the study participants had been referred for angina (96 %), were under treatment for cardiac arrhythmias and hypertension (beta blockers, 83 %), were under medication for hyperlipidemia (statins, 88 %) and were previous smokers (63 %), all high risk factors for microvascular dysfunction [29]. It is important to consider that microvascular dysfunction may have a major impact on global MBF [6, 29], which may also have affected myocardial perfusion in vessels with (no, minor or non)-obstructive CAD. The coincidental effect of microvascular dysfunction in these patients, could possibly explain the homogeneous deficiency in coronary blood flow detected with both quantitative modeling approaches.

### Study limitations

The main limitation of this study is the small population size. However, this is a pilot study to assess the feasibility of applying the DP model in this cohort of patients with known or suspected CAD. The above methods need to be assessed in larger patient cohorts to further assess their diagnostic accuracy. For perfusion CMR, a single bolus protocol was implemented to eliminate patient discomfort, similar to previous quantitative perfusion CMR studies at 1.5T [11, 19, 20] and at 3T [10]. Thus, it was impossible to assess any MBF overestimations at the specific contrast agent dose (0.05 mmol/kg) used in this study, due to arterial input function saturation issues at 3T. Our group has previously shown that the DP model was less dependent on saturation effects, although at a lower contrast agent dose (0.03 mmol/kg) [12]. However, it is currently shown here that DP modeling achieved higher sensitivity and specificity in detecting obstructive CAD in our pilot cohort. This suggests that further investigation is required to determine whether DP modeling may be a more robust method of analysis for single bolus data at 3T, compared to the Fermi model. Any possible misregistration between the actual architecture of vessel territories and the standard 16-segment model used for myocardial segmentation [14] is a methodological consideration that should not be excluded in both visual and quantitative CMR analysis. Both types of analysis can be subject to overlap of vessel territories which could in turn affect their sensitivity and/or specificity [21]. Despite this, the reference method for quantitative CMR analysis of myocardial perfusion still occurs across the three major epicardial arteries [14] and this standard type of analysis was also implemented in this study.

## Conclusions

Our analysis demonstrated that diagnostically, the DP model outperformed the standard Fermi model, in the setting of detecting hypoperfusion corresponding to haemodynamically significant stenotic vessel territories.

In per patient analysis, the diagnostic performance of DP modeling-derived MBF at stress outperformed both Fermi modeling and visual CMR analysis, whilst reaching the highest sensitivity and specificity for obstructive CAD detection in our pilot population. In the clinical setting, the haemodynamic threshold for DP-derived MBF at hyperaemia may have potential to be established as important biomarker, in order to stratify patients with at least one vessel with obstructive CAD.

## Abbreviations

CAD, coronary artery disease; CMR, cardiovascular magnetic resonance; DP, distributed parameter model; MBF, myocardial blood flow; MOLLI, modified Look Locker inversion recovery; MPR, myocardial perfusion reserve; MPR_2_, myocardial perfusion reserve of the two lowest scoring segments; PET, positron emission tomography; ROC, receiver operating characteristic

## Additional files

Additional file 1: Signal intensity and contrast agent concentration. The conversion process of signal intensity into contrast agent concentration curves is provided, using the MR pulse sequence equation. (DOCX 24 kb) Additional file 2: Fermi and distributed parameter modeling. Functions, fitted parameters and details are presented for both models. (DOCX 21 kb) Additional file 3: Microvascular characteristics. Functions of additional microvascular characteristics calculated with distributed parameter modeling. (DOCX 22 kb) Additional file 4: Microvascular characteristics values. Values of microvascular characteristics in per vessel and per patient analysis are provided. (DOCX 16 kb) Additional file 5: Bland Altman plots. Systematic bias was investigated between Fermi and distributed parameter modeling. (DOCX 268 kb)
