# Supplementary material for: Quantitative assessment of myocardial blood flow in coronary artery disease by cardiovascular magnetic resonance: comparison of Fermi and distributed parameter modeling against invasive methods
Source: J Cardiovasc Magn Reson. 2016 Sep 13;18(1):57. doi: 10.1186/s12968-016-0270-1 (PMC5022209; doi:10.1186/s12968-016-0270-1)
Supplement: Additional file 1: — Signal intensity and contrast agent concentration. The conversion process of signal intensity into contrast agent concentration curves is provided, using the MR pulse sequence equation. (DOCX 24 kb) [file 12968_2016_270_MOESM1_ESM.docx]

**Additional file 1**

The method is based on the assumption that in a region of interest, the longitudinal relaxation rate R_1_ (1/T_1_) changes linearly as a function of contrast agent concentration influx c(t) at time t multiplied by its relaxivity r_1_, according to:

$\Delta R_{1}=c(t)\cdot r_{1}$ (1)

where$\Delta R_{1}=R_{1}(t)-R_{1}(0)=\frac{1}{T_{1}(t)}-\frac{1}{T_{1}(0)}$, T_1_(0) is the native longitudinal relaxation rate (measured with MOLLI [13]) and T_1_(t) is the longitudinal relaxation rate at time t of contrast enhancement.

In equation 1, R_1_(t) is unknown and can be calculated by adapting the MR signal equation for the saturation recovery prepared single-shot FLASH sequence [11,12,17]:

(2)

where SI is the equilibrium signal intensity, Ψ is a calibration constant dependent on instrument conditions such as the receiver gain, proton density and the flip angle α. PD is the pre-pulse delay which is the time between saturation pulse and the central line of k-space, n is the number of applied pulses of flip angle α, and . TR is the time interval per phase encoding step (between repetitions of the α-radiofrequency pulses). Ψ is assumed to be constant throughout the dynamic perfusion image acquisition and can initially be calculated from equation 2 using native T_1_(0). R_1_(t) at time t of contrast enhancement can then be calculated from equation 2, using Ψ and SI values extracted from the same region of interest. Contrast agent concentration-time curves can then be calculated using equation 1.
