# Supplementary material for: Quantitative assessment of myocardial blood flow in coronary artery disease by cardiovascular magnetic resonance: comparison of Fermi and distributed parameter modeling against invasive methods
Source: J Cardiovasc Magn Reson. 2016 Sep 13;18(1):57. doi: 10.1186/s12968-016-0270-1 (PMC5022209; doi:10.1186/s12968-016-0270-1)
Supplement: Additional file 2: — Fermi and distributed parameter modeling. Functions, fitted parameters and details are presented for both models. (DOCX 21 kb) [file 12968_2016_270_MOESM2_ESM.docx]

**Additional file 2**

| Model | Fitted parameters | Fitting domain | Tissue impulse response *R* |
| --- | --- | --- | --- |
| Fermi | MBF, τ_0,_ k | Time |  |
| DP | MBF, T, T_c_, T_e_ | Laplace |  |

Fitted parameters, fitting domain and model functions for Fermi and 1-barrier 2-region distributed parameter are presented. Fitted parameters for Fermi: myocardial blood flow, τ_0_ characterized the width of the shoulder of the Fermi function and k determined the decay rate of R(t) due to contrast agent wash-out. t is the time variable. Fitted parameters for distributed parameter: myocardial blood flow, T is mean overall transit time, Tc is mean capillary transit time, Te is mean interstitial (i.e. extravascular-extracellular) transit time. Where and *f* is the frequency variable in the Fourier transformed data. DP: distributed parameter model, MBF: myocardial blood flow.

We fitted the convolution of the Fermi function with the first-pass of the arterial input function, setting the end-point at the contrast agent concentration minimum before the recirculation component begins (this range varies from patient to patient, commonly in the range between 20-35 dynamic frames). Unlike Fermi modeling, the convolution of the distributed parameter function with the entire contrast agent concentration time course of the arterial input function was fitted (i.e. 50 dynamic frames per slice).
