# Supplementary material for: Quantitative assessment of myocardial blood flow in coronary artery disease by cardiovascular magnetic resonance: comparison of Fermi and distributed parameter modeling against invasive methods
Source: J Cardiovasc Magn Reson. 2016 Sep 13;18(1):57. doi: 10.1186/s12968-016-0270-1 (PMC5022209; doi:10.1186/s12968-016-0270-1)
Supplement: Additional file 3: — Microvascular characteristics. Functions of additional microvascular characteristics calculated with distributed parameter modeling. (DOCX 22 kb) [file 12968_2016_270_MOESM3_ESM.docx]

**Additional file 3**

Functions of additional microvascular characteristics calculated for distributed parameter modeling. Myocardial plasma flow (MPF) was used to calculate permeability surface area product (PS), extraction fraction (E), extravascular-extracellular space (v_e_) and distribution volume (v_d_) and myocardial blood flow (MBF) was used to calculate intravascular space (v_b_). Hematocrit: hct.

| Microvascular characteristics | Equation |
| --- | --- |
|  |  |
|  |  |
|  |  |
|  |  |
|  |  |
|  |  |
