# Supplementary material for: Quantitative assessment of myocardial blood flow in coronary artery disease by cardiovascular magnetic resonance: comparison of Fermi and distributed parameter modeling against invasive methods
Source: J Cardiovasc Magn Reson. 2016 Sep 13;18(1):57. doi: 10.1186/s12968-016-0270-1 (PMC5022209; doi:10.1186/s12968-016-0270-1)
Supplement: Additional file 4: — Microvascular characteristics values. Values of microvascular characteristics in per vessel and per patient analysis are provided. (DOCX 16 kb) [file 12968_2016_270_MOESM4_ESM.docx]

| Microvascular characteristics | Per vessel | | Per patient | |
| --- | --- | --- | --- | --- |
|  | Group 1 | Group 2 | Group 1 | Group 2 |
| PS (mL/min/mL) | 0.73 (0.23) | 0.60 (0.20) | 0.86 (0.16) | 0.59 (0.18) |
| E (%) | 0.49 (0.07) | 0.54 (0.07) | 0.46 (0.05) | 0.53 (0.07) |
| vb (%) | 0.06 (0.03) | 0.05 (0.03) | 0.06 (0.02) | 0.04 (0.03) |
| ve(%) | 0.15 (0.06) | 0.19 (0.08) | 0.14 (0.04) | 0.19 (0.07) |
| vd(%) | 0.18 (0.06) | 0.22 (0.08) | 0.18 (0.05) | 0.21 (0.07) |

**Additional file 4**

Additional table) Mean (SD) values for permeability surface area product (PS), extraction fraction (E), intravascular space (v_b_), extravascular-extracellular space (v_e_) and volume of distribution (v_d_), estimated using the distributed parameter model. Mean values are shown for Groups 1 and 2, both in per vessel and per patient based analysis.
