# Supplementary material for: Quantitative assessment of myocardial blood flow in coronary artery disease by cardiovascular magnetic resonance: comparison of Fermi and distributed parameter modeling against invasive methods
Source: J Cardiovasc Magn Reson. 2016 Sep 13;18(1):57. doi: 10.1186/s12968-016-0270-1 (PMC5022209; doi:10.1186/s12968-016-0270-1)
Supplement: Additional file 5: — Bland Altman plots. Systematic bias was investigated between Fermi and distributed parameter modeling. (DOCX 268 kb) [file 12968_2016_270_MOESM5_ESM.docx]

**Additional file 5**


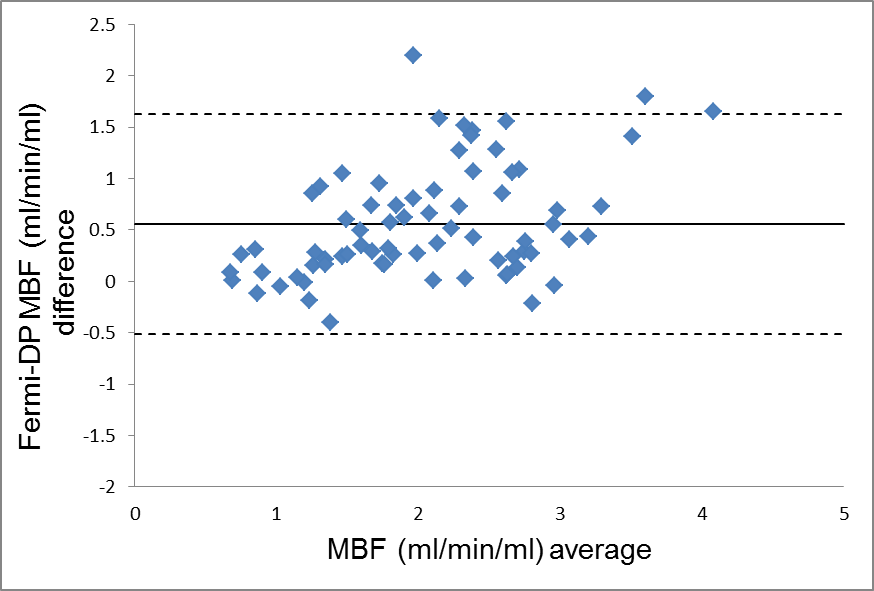

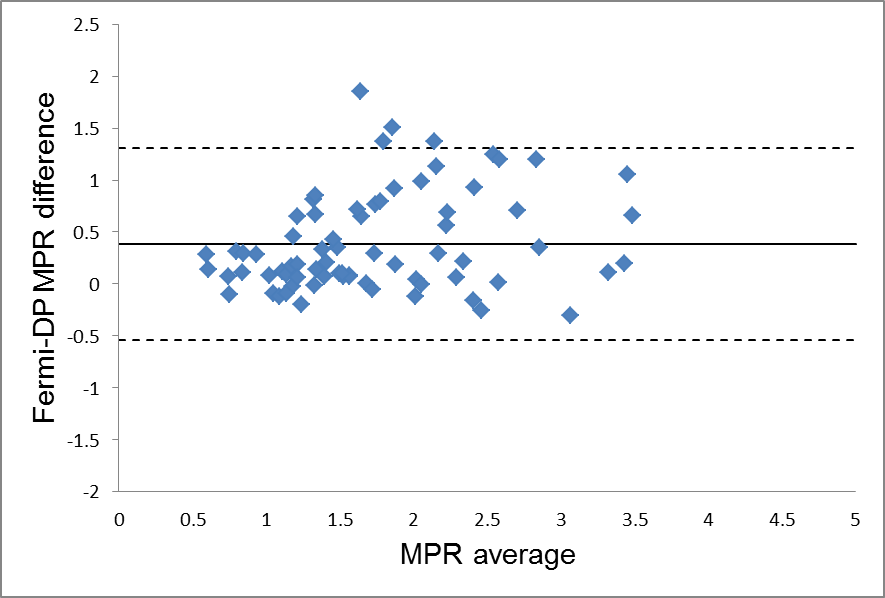

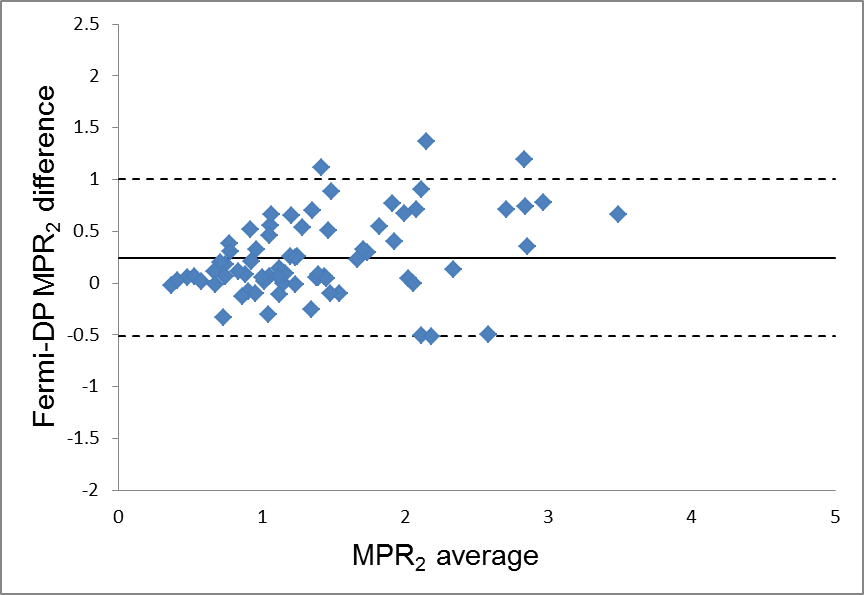


a)

b)

c)

Additional figure) Bland Altman plots showing systematic bias between Fermi versus distributed parameter modeling-derived a) MBF, b) MPR and c) MPR_2_. DP: distributed parameter modeling, MBF: myocardial blood flow at stress, MPR: myocardial perfusion reserve, MPR_2_: myocardial perfusion reserve of the two lowest scoring segments.
